# Effects of antibiotics encapsulated in hyaluronic acid hydrogels on different osteogenic cells and bacteria

**DOI:** 10.1007/s00441-026-04053-w

**Published:** 2026-02-11

**Authors:** Dimitrios Argyrakis, Ganesh N. Nawale, Oommen P. Varghese, Evangelos Mourkas, Josef D. Järhult, Nils P. Hailer, Nikos Schizas

**Affiliations:** 1 OrthoLab, Section of Orthopaedics, Department of Surgical Sciences, Uppsala University, Uppsala, Sweden; 2 Translational Chemical Biology Group, Science for Life Laboratory, Division of Macromolecular Chemistry, Department of Chemistry-Ångstrom Laboratory, Uppsala University, Uppsala, Sweden; 3 Zoonosis Science Center, Department of Medical Sciences, Uppsala University, Uppsala, Sweden

**Keywords:** Hyaluronic acid-based hydrogel, Septic arthritis, Orthopaedic infections, Osteogenic cell lines, Human-derived osteoblasts

## Abstract

Topical application of antibiotics in the treatment of orthopaedic implant-related infections can be achieved by using hyaluronic acid (HA)-based hydrogels as carriers. Our aim was to investigate potential toxic effects of a novel antibiotic-loaded hydrogel on osteogenic cells and its antibacterial effect against staphylococci. A covalently cross-linked hyaluronic acid (HA)-based hydrogel was loaded with increasing concentrations of cefuroxime and vancomycin and their release was examined by UV spectrometry. Primary human (HoBs), mouse (MoBs) osteoblasts, or SaoS-2 cells were either exposed to the drug-loaded hydrogel or to antibiotics alone, followed by assessment of cell metabolism and proliferation. Antibacterial effects were evaluated against *Staphylococcus aureus* (*S. aureus*) and *Staphylococcus epidermidis* (*S. epidermidis*). Increasing concentrations of antibiotics did not affect cell metabolism in any osteogenic cell type, whereas cell proliferation remained unaltered in MoBs, was significantly reduced in SaoS-2, and was stimulated in HoBs. Cultures of MoBs and HoBs tolerated higher concentrations of vancomycin than SaoS-2. Antibiotic-loaded hydrogels did not exert toxic effects on HoBs. After 24 h, 16.8% of vancomycin and 70.8% of cefuroxime were released from the hydrogel. Cefuroxime-loaded hydrogels significantly inhibited growth of *S. aureus* but not of *S. epidermidis*, while vancomycin-loaded hydrogel had scarce effects on *S. epidermidis*. Loading HA-based hydrogel with antibiotics does not harm osteoblasts at clinically relevant concentrations but inhibits bacterial growth. Higher loading of vancomycin may be required due to its slow release while cefuroxime is released more rapidly. A resorbable, antibiotic-loaded hydrogel may be used for implant-related infections in orthopaedics.

## Introduction

Septic arthritis and implant-related infections are associated with high morbidity and mortality rates, extended hospitalisation, long-term use of antibiotics, and frequent re-admissions (Badia et al. 2017; Patel et al. 2017; Parisi et al. 2017; Akindolire et al. 2020; Piednoir et al. 2021), with staphylococci being the most frequent microbial agent (Dubost et al. 2002; Linke et al. 2022).

Treatment of implant-related infections usually involves multiple surgeries and systemic use of antibacterial agents, but some of these have limited access to the infection site (Acosta-Olivo et al. 2021; Balato et al. 2021; Kurapatti et al. 2022; Russo et al. 2022; Walter et al. 2022). In most cases, high plasma antibiotic concentrations are needed to achieve tissue and joint concentrations that are effective towards various pathogens due to biofilm formation as well as variable pharmacokinetics of each antibiotic(Schwameis et al. 2017).

Hyaluronic acid (HA)-based hydrogels have been studied for their potential as local drug carriers (Patterson et al. 2010; Burdick and Prestwich 2011; Xu et al. 2021). They can reduce or prevent bacterial colonisation of and biofilm formation on orthopaedic implants and have been used both in the prevention and treatment of periprosthetic joint infection (PJI) (Romanò et al. 2015; Malizos et al. 2017; De Meo et al. 2021; Carpa et al. 2022). This approach is however not straightforward, since the cross-linking reaction of hydrogels shaped in situ should not be associated with the formation of side products that are toxic to the surrounding tissue (Hilborn 2011). Additionally, the concentration of loaded antibiotics must be carefully controlled because a considerable amount of antibiotic may bind to the hydrogel, negatively affecting release rates.

Therefore, apart from the nature and biocompatibility of the hydrogel, the efficacy and toxicity of a range of antibiotic concentrations need to be evaluated with and without HA-based hydrogels in order to design novel biomaterials loaded with antibiotics. It is expected that the actual dose of released antibiotics to the surrounding tissue will be lower than the one loaded in the gel; therefore, the tolerance of osteogenic cells was tested in concentrations far above the clinically relevant concentrations. This is because the hydrogels should be loaded with high doses of antibiotics in order to release a satisfactory concentration in the surrounding tissue.

In the present study, we primarily investigated the effect of two antibiotics that are frequently used in the prophylaxis or treatment of implant-related infections. Cefuroxime and vancomycin, although not typically co-delivered as a fixed combination, are both frequently used in the treatment of implant-related infections, making them suitable for the assessment of HA-based hydrogel compatibility with antibiotics of different classes and release characteristics. The antibiotics were firstly delivered in various concentrations on different osteogenic cell cultures. Secondly, we reviewed whether the HA-based hydrogel, alone, or loaded with antibiotics, exerted toxic effects or impaired osteogenic cell activity, and the antibiotic release profile of antibiotic-loaded hydrogel was examined. Lastly, we studied the antibacterial action of the crosslinked, antibiotic-loaded HA-based hydrogel on *S. aureus* and *S. epidermidis*.

## Materials and methods

### Experimental plan

All experiments were conducted under aseptic conditions using sterile instruments after approval from the local committee for research and ethics and the local committee for animal welfare (dnr: 2020–04462, 5.8.18–00683/2017 and 5.8.18–16,672/2019). Our study involved three major experimental settings as described below. Each experimental group involved 6 biological replicates, suggested after power analysis.

(i) Toxicity experiments were performed on mouse-derived osteoblasts (MoBs), SaoS-2 cells, and human-derived osteoblasts (HoBs). Primary cultures of MoBs, SaoS-2, and HoBs were exposed to increasing concentrations of cefuroxime or vancomycin. The experiment was conducted in three technical replicates. The cells were incubated for 48 h at 37 °C and 5% CO_2_ before the evaluation of metabolic activity and proliferation. Cell metabolism was examined by alkaline phosphatase (ALP) assay normalised to lactate dehydrogenase (LDH) assay, and cell proliferation was examined by the CellTiter 96® AQueous One Solution Cell Proliferation (MTS) assay.
(ii) HA-based hydrogels loaded with antibiotics in a range chosen from the above experiment were applied for 48 h on HoBs. Hydrogel cytocompatibility assays were performed using HoBs, as these cells most closely represent the physiological osteogenic response in vivo. SaOS-2 cells, although useful for comparing antibiotic susceptibility across cell types, are a tumour-derived immortal cell line and do not accurately reflect the phenotype or behaviour of primary osteoblasts. For this reason, only HoBs were selected for direct interaction studies with the HA-based hydrogel. Cell metabolism was assessed by ALP assay normalised either to values from cell cultures in the absence of hydrogel or to values from hydrogel in the absence of cells. The proliferative activity was assessed by MTS. Additionally, a time-lapse experiment of a 7-day incubation was performed to evaluate the effects of antibiotics loaded in HA-based hydrogels on HoBs after a longer period of incubation. The time-lapse experiment was assessed by MTS.
(iii) Application of antibiotic loaded HA-based hydrogels on bacterial cultures to evaluate the antibacterial effect, similar to the disc diffusion test. Evaluation was performed by quantitative measurements of the inhibition zone.

### Osteogenic cell cultures

Primary cultures of MoBs were isolated from five 3-week-old C57Bl6 mice as previously described (Taylor et al. 2014; Chevalier et al. 2021). No grouping was performed prior to dissections, and they were sacrificed by decapitation. The limbs were removed from the body by cutting with sharp scissors, taking care to preserve as much of the limb as possible. The femora and tibiae were extracted with sterile tools, and the skin, tendons, and muscles were removed by scissors and with a sterile gauze. The epiphyses were cut off, and the bone fragments were placed into a flat-bottomed 5-mL tube. The bone marrow was flushed several times with a 1-mL syringe with a 25-G needle in a 1.5-mL Eppendorf tube with 1 mL medium until the bone became completely devoid of bone marrow. Each bone was cut with sterile scissors into small pieces with a length of 2 mm and put them into a 25-cm^2^ flask containing 6-mL culture medium (alpha minimal essential medium [MEM; GE healthcare, Sweden], 10% FBS [Sigma-Aldrich, Sweden] and 2 mmol/L L-glutamine).

Primary cultures of HoBs were isolated from five human donors undergoing hip or knee replacement, as previously described (Gundle and Beresford 1995; Gundle et al. 1998; Perpétuo et al. 2019). None of the subjects had any metabolic bone disease or rheumatoid arthritis. The bone tissue was removed from the hip or knee joint and transported to the lab in sterile α-MEM (GE Healthcare) the same day. Soft connective tissue and coagulated marrow from the outer surfaces of the bone were detached by scraping in a sterile petri dish containing 5–10 ml of PBS. Bone cutters were used to carefully cut the bone into 2-mm fragments. The diced bone fragments were placed in 25-cm^2^ flasks containing α-MEM (GE Healthcare) supplemented with 10% FBS (Sigma-Aldrich), 50 U/mL penicillin, and 50 mg/L streptomycin (PeSt), and 2 mmol/L L-glutamine (SVA, Uppsala, Sweden).

The SaoS-2 cells (Primary osteogenic sarcoma human, catalogue nr. 89,050,205-1VL) were purchased from Sigma (ECACC) and incubated in 25 cm^2^ cell culture flasks at 37 °C in a 5% CO_2_ enriched atmosphere. The cell medium was replaced once a week.

All the experimental series involving MoBs, HoBs, and SaoS-2 were conducted based on the same pattern: the medium was refreshed weekly until the cells were approximately 90% confluent (4–6 weeks). Twenty-five-square centimetre flasks were used for passage 1 and 2, while from passage 3, cells were cultured in 75-cm^2^ flasks. The cultures were undisturbed for 7 days to ensure the outgrowing cells adhered to the tissue culture plastic. Cells became confluent after 4–8 weeks. Prior to the experiment, cells were washed with Phosphate-buffered saline (PBS), trypsinised, and the medium was changed to α-MEM containing L-Glutamine (2 mM), ribonucleosides (40 mg/L), deoxyribonucleosides (41 mg/L), and 10% FBS. The prepared cells were seeded at 10,000 cells/100 μL in 96-well plate, each well containing 100 μL, and incubated for 24 h to achieve cell adhesion.

To relate antibiotic concentrations in our experiments to those targeted in clinical practice, we defined a clinically relevant concentration (CRC) as a concentration where the antibiotics are likely to have a clinical effect on a susceptible bacterial strain. CRCs were defined as 2 mg/L for vancomycin based on clinical breakpoint against *S. aureus* (https://www.eucast.org/clinical_breakpoints) and 0.5 mg/L for cefuroxime based on susceptibility of a reference strain of *S. aureus* (Andrews 2001).

After 24 h, when cell-surface adhesion was achieved, antibiotic solutions in increasing concentrations up to 50 × CRC were introduced and the cultures were incubated for an additional 48 h. The control group contained osteoblasts and culture medium in the absence of antibiotics.

### Cell metabolic activity and proliferation

Cell metabolism was studied by ALP and LDH assays while cell proliferation was studied by MTS assays.

Primary osteoblasts were seeded at a density of 10,000 cells/well in 96-well plates. The osteoblasts were washed with 100 μL of PBS 48 h after antibiotic administration. The cells were obtained by addition of 13.5 μL of Lysis Buffer and agitation on Microplate Shaker for 15 min at 1200 rpm. Afterwards, 36.5 μL of Milli-Q water was added to the samples.

ALP was measured by colorimetric ALP Sigma assay kit P7998. The samples were mixed with 100 μL of p-nitrophenyl phosphate substrate and incubated at 37 °C and 5% CO_2_. The absorbance at 405 nm was read at the microplate reader after 60 min.

An LDH-assay was used to measure intracellular LDH of osteoblasts as an indication of cell viability. The osteoblasts were prepared according to the ALP-assay, but with the following modifications. After the addition of Milli-Q water, the LDH Sigma assay kit TOX7 was used to monitor in vitro cytotoxicity of osteoblasts after antibiotic treatment. The LDH mixture was obtained by combining equal volumes of the LDH Assay Substrate Solution (L2402), LDH Assay Cofactor Preparation (L2527), and LDH Assay Dye Solution (L2277). The 100μL of LDH mixture was loaded to each well and incubated covered and at room temperature. Data were collected at the absorbance of 490 nm and 690 nm by the microplate reader after 60 min.

The MTS assay was performed to assess the proliferative activity of viable osteoblasts. The 20 μL CellTiter 96® Aqueous One Solution Cell Proliferation Reagent was loaded to each well of the 96-well plate containing osteoblasts in 100 μL of the medium. The osteoblasts were incubated at 37 °C and 5% CO_2_, and absorbance at 490 nm was recorded after 120 min.

### HA hydrogel encapsulated with antibiotics

To develop antibiotic-loaded hydrogels, we employed dynamically cross-linked hydrogel chemistry that consists of two components forming a semisolid hydrogel within seconds after mixing. For this purpose, HA-aldehyde and HA-hydrazide components were utilised in a click-type reaction to produce the HA hydrogel by forming a hydrazone linkage (Oommen et al. 2013). The components were dissolved in culture medium at a concentration of 18 mg/ml. Antibiotics were dissolved in the HA-aldehyde component while HoB solutions of 10 000 cells/20 μL were added in the HA-hydrazide component. Synthesis of acidic HA-aldehyde and HA-hydrazide derivatives was performed following previous publications (Wang et al. 2013; Yan et al. 2018). Antibiotic concentrations that were not toxic for HoBs (5 ×, 15 ×, and 30 × CRC) were integrated with the novel HA hydrogel to investigate whether it impairs osteogenic cell activity when it is loaded with antibiotics. Two control groups were studied, one with the presence of cells and hydrogel in medium in the absence of antibiotics, and the second one in the absence of HA hydrogel and antibiotics. For normalisation a control group with hydrogel and medium but without cells or antibiotics was used. A TS analysis was carried out after 2, 48, 72, and 168 h to examine the potential impact on cell proliferative activity over time. For this experiment, we compared our cross-linked HA hydrogel to a commercially available HA hydrogel already used in clinical practice (Healon 5®, Abbott, Uppsala, Sweden), after incorporating antibiotics to evaluate the potential inhibition of cell activity.

### Release of antibiotics from integrated HA hydrogel

HA-aldehyde and HA-hydrazide derivative were prepared and quantified following previously reported methods where the degradation pattern of the hydrogel was studied (Oommen et al. 2013; Wang et al. 2013). Hyaluronic acid (HA, 200 kDa) was purchased from Lifecore Biomedical, LLC (Chaska, MN). A Lambda 35 UV/Vis spectrophotometer from PerkinElmer instruments was used for spectroscopic analysis. HA-hydrazide (~ 7% modification) and HA-aldehyde (~ 12% modification) were separately dissolved in culture media to reach a concentration of 20 mg/ml. The hydrogel was prepared (80 µL) by mixing equal volumes of HA-hydrazide and HA-aldehyde solution and antibiotic (5 µL from a stock of 12.5 mg/mL loaded in a gel volume of 80 µL corresponds to a final antibiotic concentration of 780 µg/mL) and different salts (10 µL from 1 M stock were added) to achieve final reaction volume of 200 µL with culture media (25–40 µL).

Scaffolds of integrated HA hydrogel were incubated in culture media alone or in the presence of 50 mM of either NaCl or MgCl_2_ to induce release. The release kinetics of each antibiotic from the hydrogel was examined by A280 nm UV-spectrometry. The concentration of antibiotics released in the medium was measured after 1, 4, 24, 48, and 144 h.

### Bacterial strains

Strains of *S. aureus* and *S. epidermidis* were used in this study. These strains were isolated from patients with PJI and identified to species levels by routine methods in the clinical microbiology laboratory at our University Hospital. For each use, the bacterial strains were thawed at room temperature for approximately 5 min and then plated onto blood agar with 5% horse blood, using a 1 µL loop (Sarstedt, 86.15767.050). A 0.5 McFarland suspension of each bacterial isolate was used as inoculum and spread using a sterile cotton swab onto blood agar plates. The plate was incubated for 24 h at 37 °C to allow bacterial growth.

### Bacterial inhibition

HA hydrogels integrated with antibiotics up to a concentration of 30 × CRC were applied to established cultures of *S. aureus* or *S. epidermidis* to examine the effect on bacterial growth by the zone of inhibition test. One *S. aureus* and one *S. epidermidis strain*, both isolated from patients with periprosthetic joint infection (PJI) at Uppsala University Hospital, were used in this experiment. For each strain, six biological replicates were performed. Moreover, HA hydrogels alone were similarly examined for their potential to inhibit bacterial growth.

Following bacterial plating as described above, vancomycin and cefuroxime loaded in the HA-based hydrogel in concentrations of 5 × and 30 × CRC were applied on the plates. Hydrogels without antibiotics served as control groups. The plates were then incubated at 37 °C for 24 h to allow for bacterial growth. To evaluate bacterial inhibition, we measured the width of the clear zone around the droplet where no bacterial growth was observed.

### Statistical analysis

Data were described using means and standard deviations. For comparisons of means between groups, one-way ANOVA followed by pairwise *t*-test using the Bonferroni correction was carried out for data that were normally distributed and did not violate the assumption of homogeneity of variance, as ascertained by Levene’s test. Otherwise, the non-parametric Kruskal Wallis and Mann Whitney *U*-tests were used. In the time-lapse experiment, planned contrasts were used. *p*-values < 0.05 were considered statistically significant. The software SPSS (IBM) and R (version 4.2.2) were used.

## Results

### Antibiotic toxicity on osteogenic cells

**Fig. 1:**
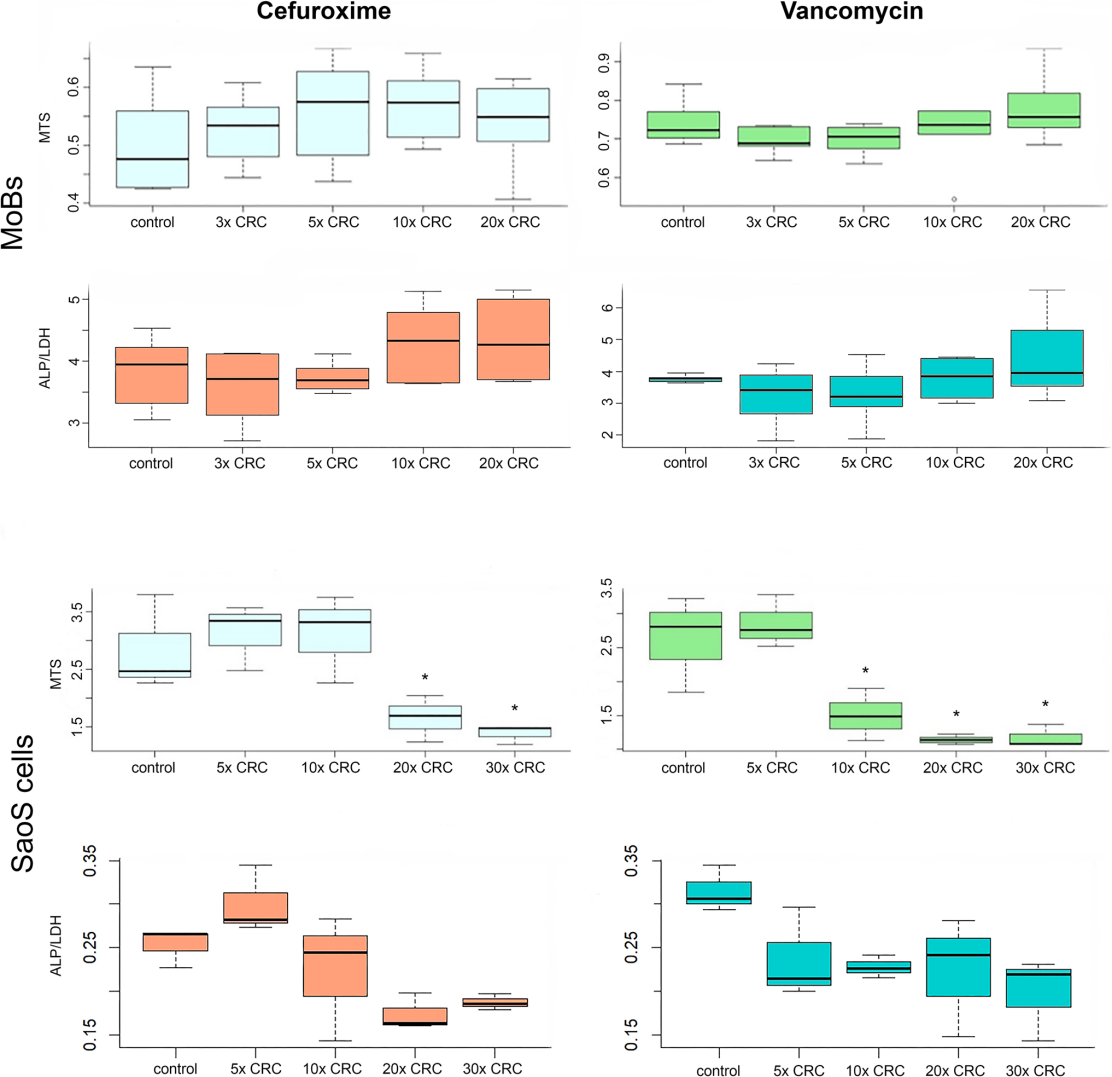
Graphs showing metabolic activity (ALP/LDH) and proliferation (MTS) of osteogenic cell cultures after a 48-h incubation in increasing concentrations of antibiotics. The culture medium in the control groups was free from antibiotics. Asterisks denote statistically significant difference (*p* < 0.05) compared to the respective control group

Various concentrations of antibiotics differentially affected the examined osteogenic cell cultures. MoBs were not affected by either cefuroxime or vancomycin concentrations of up to 20 × CRC in terms of cell proliferation and metabolic activity measured by ALP/LDH assay and MTS (Fig. 1).

**Fig. 2:**
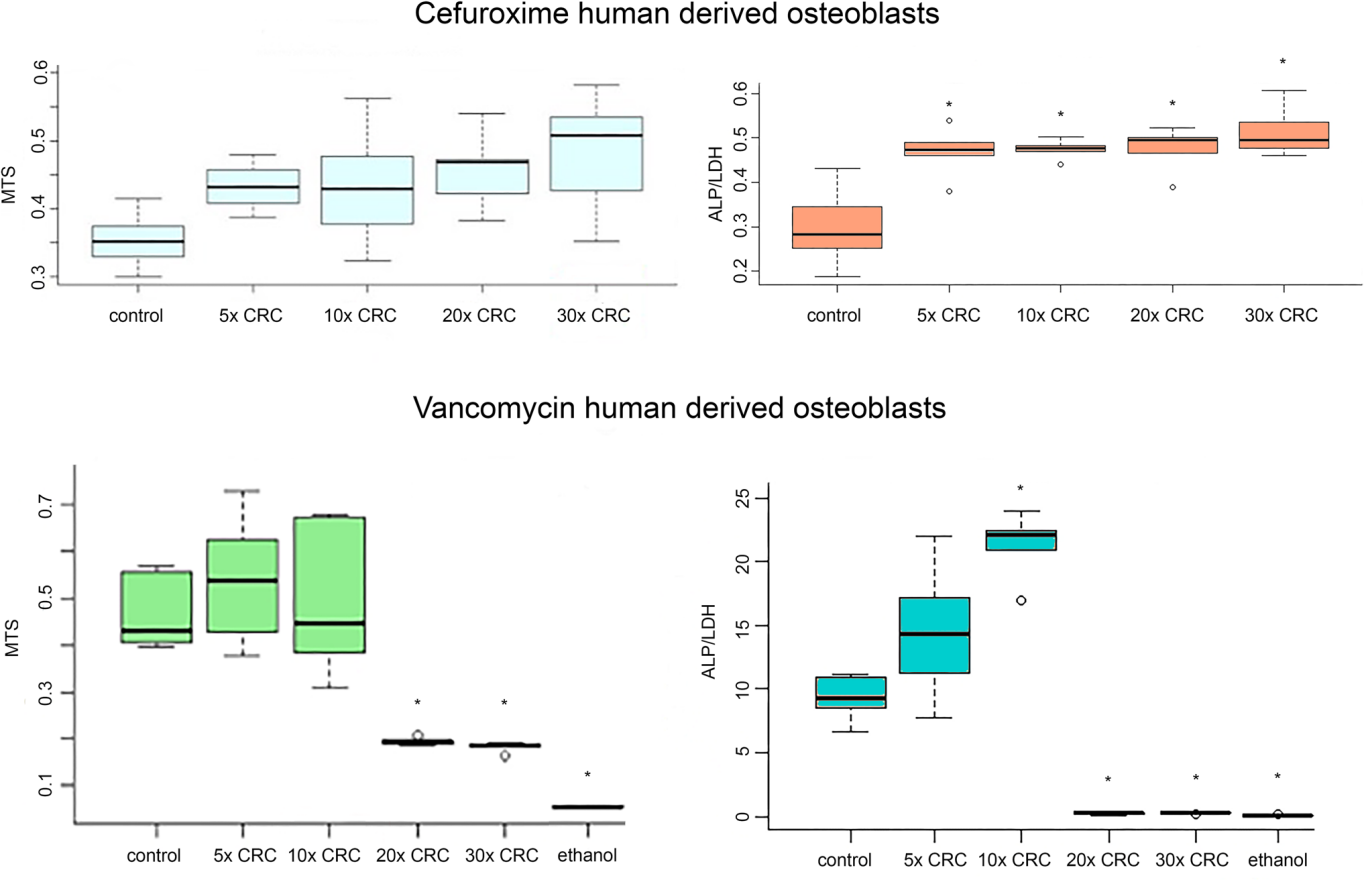
Graphs showing metabolic activity (ALP/LDH) and proliferation (MTS) of HoBs cultures after a 48-h incubation in increasing concentrations of antibiotics. The culture medium in the control groups was free from antibiotics. The group ‘ethanol’ involved the presence of ethanol in the culture medium as a negative control. Asterisks denote statistically significant difference (*p* < 0.05) compared to the respective control group

In HoBs, cefuroxime concentrations of up to 30 × CRC stimulated ALP/LDH metabolic activity, whereas concentrations of up to 20 × CRC did not affect osteoblast proliferation. Vancomycin concentrations of up to 10 × CRC did not affect osteoblast cultures, as measured by ALP/LDH-activity and MTS. Concentrations higher than 10 × CRC significantly reduced cell proliferation, measured by MTS, and ALP/LD metabolic activity (Fig. 2).

In SaoS-2 cells, Cefuroxime concentrations of up to 10 × CRC did neither affect ALP/LDH activity nor proliferation. When the concentration of cefuroxime was increased to 20 × and 30 × CRC, ALP/LDH activity was still not affected; however, cell proliferation in terms of MTS was reduced compared to controls (Fig. 1). SaoS-2 cells maintained in the presence of vancomycin at concentrations higher than 5 × CRC clearly showed a decline in cell proliferation, as assessed by MTS, while ALP/LDH activity was not affected (Fig. 1).

### Effects of antibiotic-loaded HA hydrogels on HoBs

**Fig. 3:**
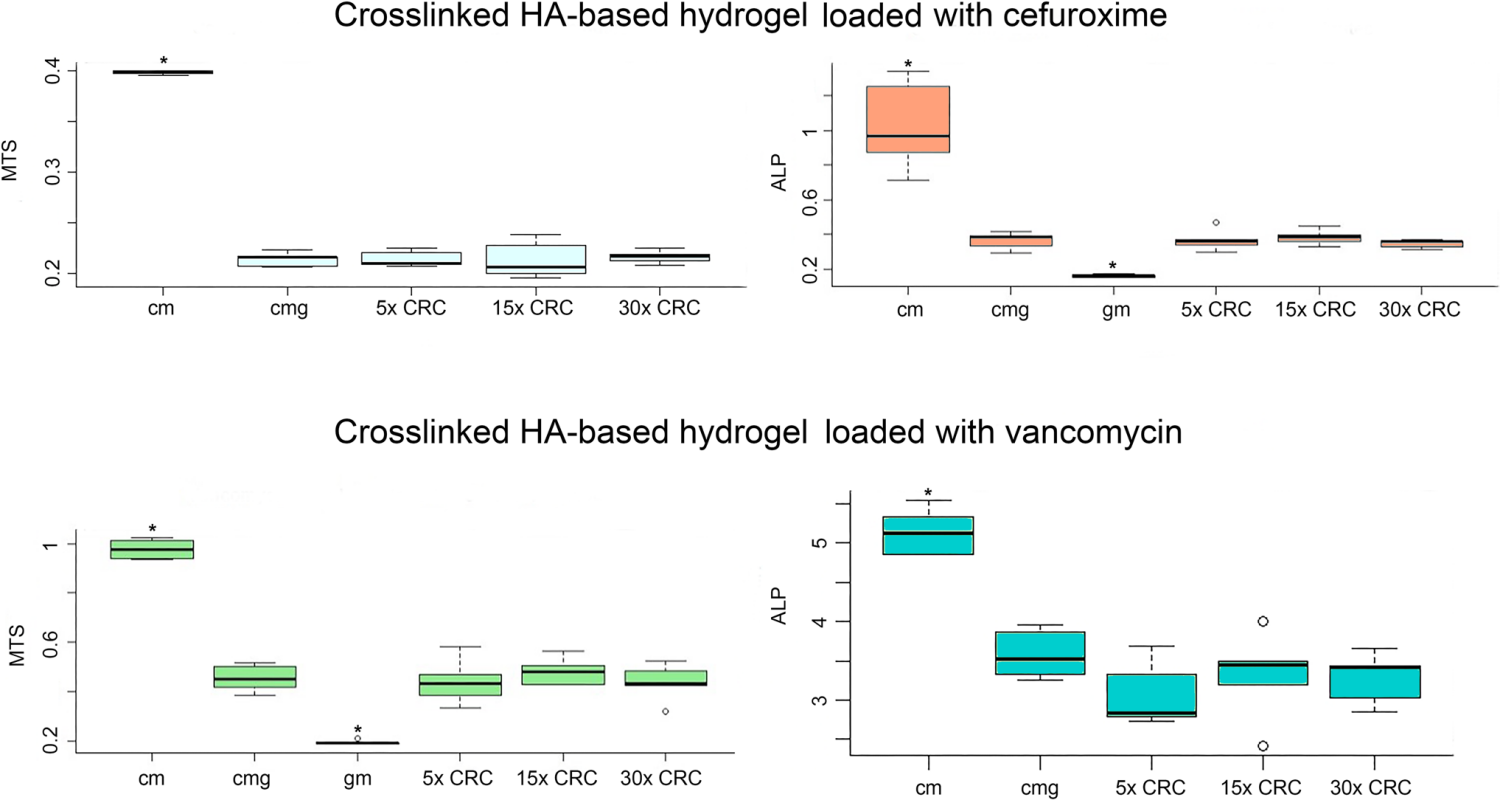
Graphs showing metabolic activity (ALP/LDH) and proliferation (MTS) of HoBs cultures maintained in HA hydrogel scaffolds integrated with increasing concentrations of either cefuroxime or vancomycin. The groups ‘cells medium (cm)’, ‘cells medium gel (cmg)’, and ‘gel medium (gm)’ were free from antibiotics. The group ‘cm did not involve the presence of hydrogel, while ‘gm’ did not involve cell cultures, and both were used for normalisation. The group ‘cmg’ served as a control group in the absence of antibiotics. Asterisks denote statistically significant difference (*p* < 0.05) compared to the respective control (cmg) group

HA-based hydrogels, loaded with cefuroxime concentrations of up to 30 × CRC, did not affect human osteoblast activity and proliferation compared to osteoblast cultures incubated in HA-based hydrogels without antibiotics (Fig. 3).

**Fig. 4:**
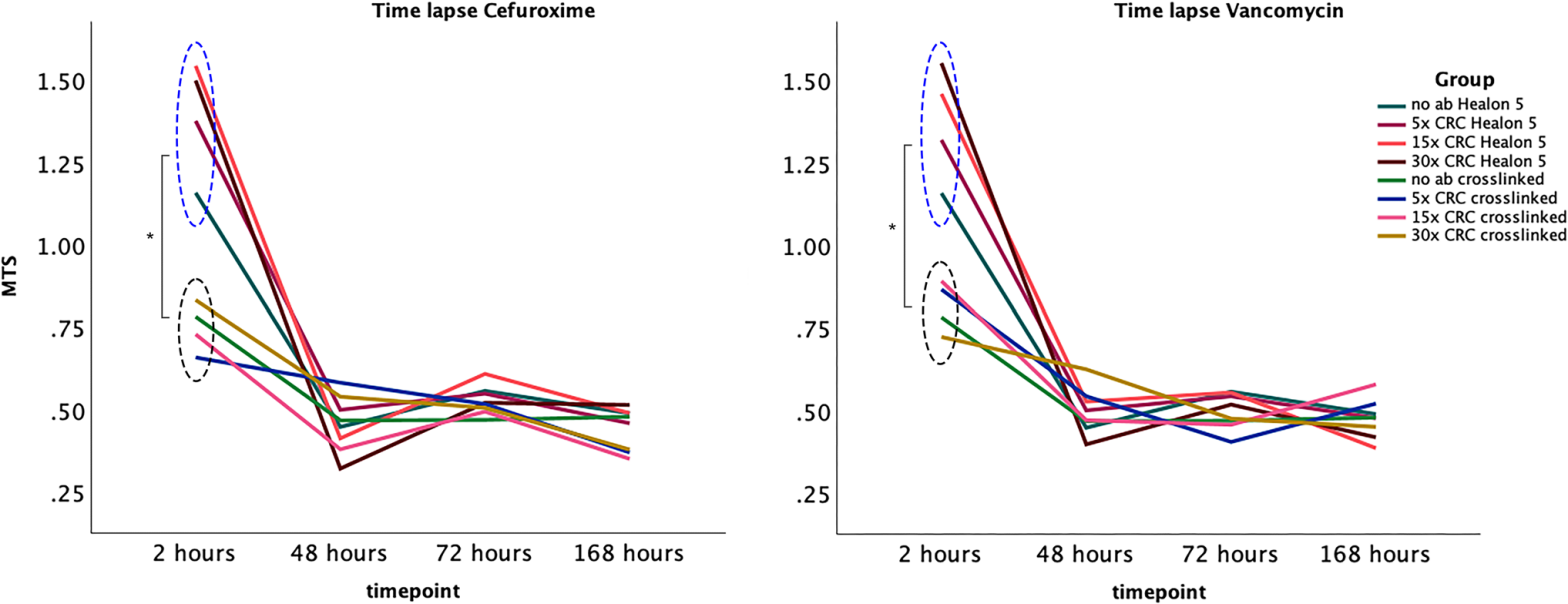
Graphs showing the proliferation rate (MTS) of HoBs after a time-lapse experiment up to 168 h. The cell cultures were incubated in hydrogel scaffolds integrated with increasing concentrations of vancomycin and cefuroxime. A non-crosslinked HA-based hydrogel (Healon 5, Abbott, Sweden) was used as control. At the onset of the experiments, the proliferation rate was significantly higher in the cultures maintained in the non-crosslinked hydrogel (blue dashed ellipse) compared to the crosslinked hydrogel (black dashed ellipse). However, at later time points, the proliferation rate normalises between the groups, and there are no significant differences up to 168 h of incubation. The addition of increasing concentrations of antibiotics did not seem to affect the proliferation rate at any time point (* *p* < 0.05)

Additionally, HA-based hydrogels integrated with vancomycin did not affect cell activity and proliferation compared to human osteoblast cultures incubated in HA-based hydrogels without the presence of antibiotics (Fig. 3). Furthermore, a 7-day extended time-lapse analysis was performed to examine the potential impact on cell activity and proliferation rate measured by MTS, and comparison was made to a non-cross-linked HA hydrogel available for use in ophthalmic surgery (Healon 5®). At the onset of the experiment (2-h timepoint), the crosslinked HA-based hydrogel initially seemed to hamper the osteoblast proliferation (Fig. 4). However, after 48 h, when the cultures were settled, the proliferation pattern was similar between the two compared hydrogels (Fig. 4).

### Release kinetics of the HA hydrogel

**Fig. 5:**
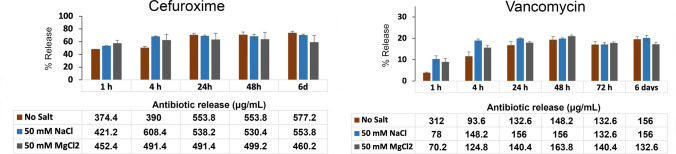
Antibiotic release rate from the integrated HA hydrogel after incubation in culture medium. The release rate was studied with spectrometry and expressed as % of the original concentration. Cefuroxime has a quite rapid release from the hydrogel, with almost 70% of the concentration released in the first 24 h. Vancomycin, on the other hand, has a slow release, with the vast amount of the antibiotic still encapsulated in the hydrogel after 6 days of incubation

After 24 h, 16.8% of vancomycin and 70.8% of cefuroxime were released from the HA hydrogel and the release was stabilised after that timepoint (Fig. 5). The release of vancomycin was induced in the presence of NaCl (19.9%) and in the presence of MgCl2 (17.9%) while the release of cefuroxime was decreased (69.1% and 63.2%, respectively). However, none of these differences were statistically significant.

### Antibiotics and HA hydrogels on bacterial cultures

**Fig. 6:**
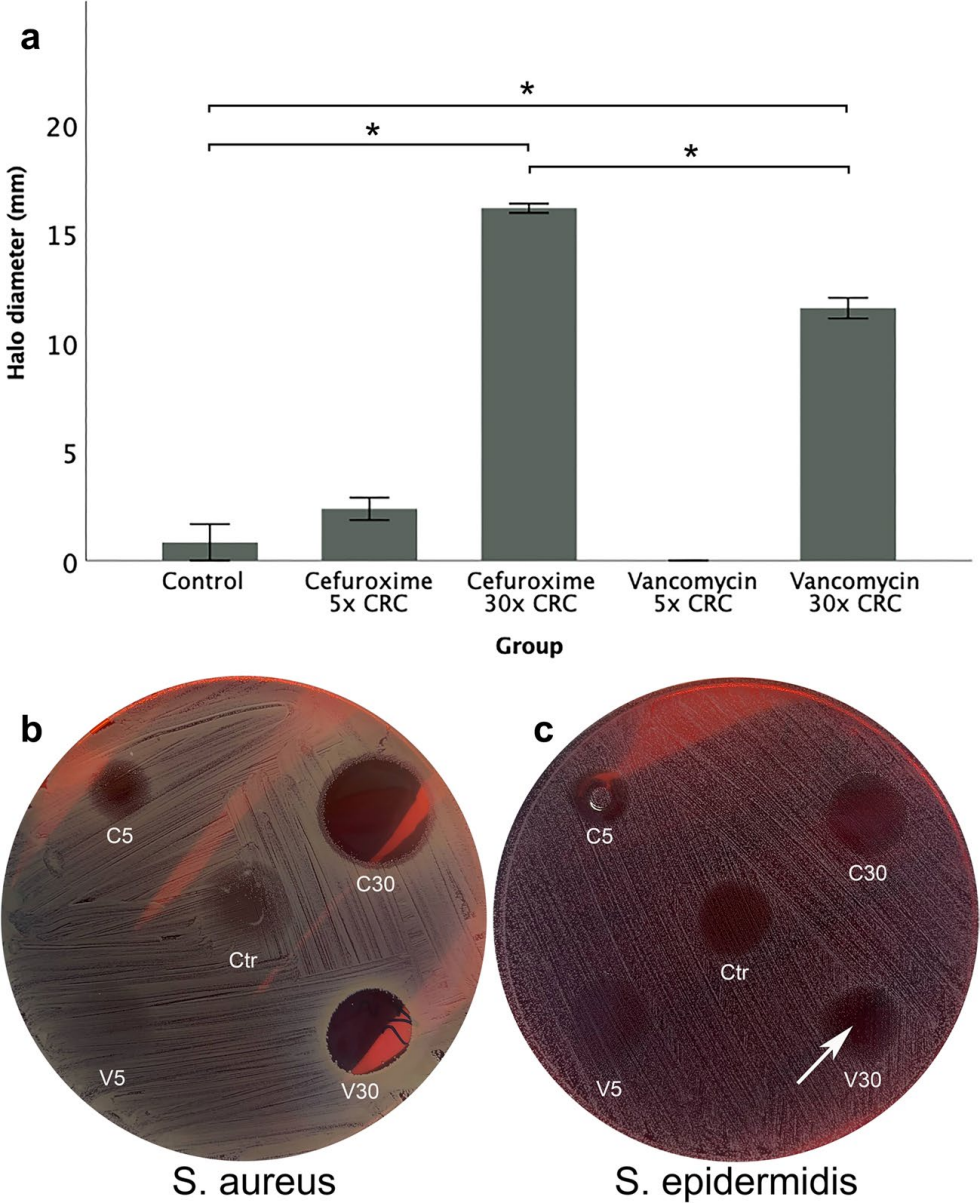
**a**–**c** The graph shows inhibition of S. aureus expressed in zone widths after exposure to cefuroxime and vancomycin (**a**). The concentration of 30 × CRC of both cefuroxime and vancomycin integrated in HA-based hydrogels inhibited *S. aureus* growth significantly compared to HA-based hydrogels alone. The zone of inhibition related to 30 × CRC cefuroxime was slightly larger than the one corresponding to 30 × CRC vancomycin (**a**, **b**). Regarding *S. epidermidis*, inhibition of growth was only observed in the 30 × CRC vancomycin group, and it was rather scarce, with no measurable halo diameter but a clear inhibition of growth within the epicentre of the hydrogel (**c**) (* *p* < 0.05, Ctr = control, C5 = 5 × CRC cefuroxime, V5 = 5 × CRC vancomycin, C30 = 30 × CRC cefuroxime, V30 = 30 × CRC vancomycin)

A clear inhibition of *S. aureus* was observed after application of HA-based hydrogels loaded with 30 × CRC cefuroxime as well as hydrogels loaded with 30 × CRC vancomycin (Fig. 6). The halo width that corresponded to 30 × CRC cefuroxime was 16.2 ± 2 mm, and it was significantly higher compared to gel alone (0.8 ± 0.8 mm, *p* < 0.001) and to 30 × CRC vancomycin (11.6 ± 0.5 mm. *p* < 0.001, Fig. 6a). The halo diameter that corresponded to 30 × CRC vancomycin was also significantly higher than the control (*p* < 0.001, Fig. 6a). Hydrogels loaded with 5 × CRC cefuroxime as well as vancomycin did not display a significant inhibition (Fig. 6a, b).

In the case of *S. epidermidis*, growth inhibition was only observed in the 30 × CRC vancomycin group, and it represented a scarcer distribution of bacterial colonies within the epicentre of the hydrogel compared to the rest of the groups, rather than a measurable halo width (Fig. 6c).

## Discussion

This study supports critical benefits regarding the use of cefuroxime- and vancomycin-loaded hydrogels in concentrations exceeding clinically relevant thresholds, demonstrating a dual advantage, effectively inhibiting bacterial growth while maintaining osteoblast viability and proliferation. It advances previous work on antibiotic-loaded HA hydrogels by combining antibacterial assessment with a detailed evaluation of osteogenic cell cytocompatibility across several cell types, using clinically relevant antibiotic concentrations. In addition, we examine a covalently cross-linked HA hydrogel formulation whose interaction with osteogenic cells has not previously been characterised. The release dynamics of the aforementioned antibiotics differed significantly; therefore, vancomycin may require higher loading concentrations in clinical praxis due to its slower release profile, whereas cefuroxime exhibited rapid and efficient release.

### Local application of antibiotics

The local application of antibiotics has gained prominence in orthopaedic surgery as a means to achieve high local antibiotic concentrations, minimise systemic toxicity, and effectively reduce infection rates (Cancienne et al. 2015; Peng et al. 2021; Vaida et al. 2022; Kruse et al. 2023). However, topical application of antibiotics is, in many centres, not yet recommended routinely, at least not before the conduction of multicentre prospective trials demonstrating a clear reduction in infection rates (Wong et al. 2021). Vancomycin has been widely used due to its potent activity against Gram-positive bacteria, particularly methicillin-resistant *S. aureus* (MRSA) and methicillin-resistant coagulase-negative staphylococci (MRCoNS), and its cost-effectiveness. Cefuroxime, a second-generation cephalosporin, is another frequently used antibiotic for prophylaxis in orthopaedic surgery. Its wide antimicrobial spectrum, resistance to some beta-lactamase degradation, excellent bone penetration, and low incidence of adverse effects make it particularly suitable for infection prevention.

The antibiotic concentrations applied in this study that ranged between 2 and 100 mg/L in the case of vancomycin and 0.5–25 mg/L for cefuroxime were selected to reflect the sustained levels that osteogenic cells are likely to encounter after the initial burst release associated with local antibiotic delivery. Although very high peak concentrations, often in the mg/mL range, have been reported in vivo following topical or cement-based administration, these concentrations decrease rapidly within the first hours, and levels in the low µg/mL range typically predominate during the subsequent 24–72 h (Belt et al. 2000; Li et al. 2024). Our release data similarly demonstrated partial early release, consistent with this post-burst exposure window. In addition, the concentrations tested were chosen to exceed CRC, because a considerable proportion of the antibiotic is expected to bind to the hydrogel matrix and adjacent biomaterial surfaces, reducing the freely bioavailable fraction reaching surrounding cells. Thus, the tested doses represent a conservative, safety-oriented exposure range.

Previous studies reporting cytotoxic effects of vancomycin or cefuroxime on osteogenic cells have generally used substantially higher concentrations than those employed here (Edin et al. 1996; Liu et al. 2018; Hanson et al. 2021). Vancomycin toxicity is most often observed above 1–5 mg/mL (Edin et al. 1996; Antoci et al. 2007), while cefuroxime-induced cytotoxicity typically occurs above 250–500 mg/L (Miclau et al. 1993). The concentrations used in our study fall well below these established cytotoxicity thresholds, which is consistent with our observation that neither antibiotic impaired osteoblast metabolism. Our findings therefore complement earlier work by confirming the absence of toxicity at lower, clinically plausible concentrations and support the suitability of HA-based hydrogels for local antibiotic delivery without compromising osteogenic cell viability.

### Hyaluronic acid hydrogels as carriers for antibiotics

HA, or hyaluronan, is a glycosaminoglycan (GAG) with repeating disaccharide units (Fakhari and Berkland 2013; Caon et al. 2021). HA plays an essential role in the composition and structure of the extracellular matrix (ECM) (Zustiak et al. 2013), regulates cell adhesion, migration, and morphogenesis, adjusts cell proliferation and differentiation (Lam et al. 2014), and has important effects on the development, organisation, or remodelling of tissue, angiogenesis process, modulation of inflammation, and wound healing (Bastow et al. 2008; Collins and Birkinshaw 2013).

HA hydrogels are desirable biomaterials for medical applications due to their high-water content, good mechanical properties, non-immunogenicity, promising biocompatibility, versatility, sufficient biodegradability along with high permeability to oxygen, nutrients, and other water-soluble metabolites (Hanjaya-Putra et al. 2013; Pang et al. 2014). Furthermore, HA-based hydrogels are reported to inhibit cell attachment due to their hydrophilic and polyanionic nature, since cells were more willing to selectively adhere to neutral, hydrophobic, or polycationic surfaces of materials. In recent years, hyaluronic acid (HA) hydrogels have been studied for their potential as drug carriers (Patterson et al. 2010; Burdick and Prestwich 2011; Highley et al. 2016; Gaetano et al. 2018; Xu et al. 2021) since the use of antibiotic-loaded HA hydrogels can reduce or prevent bacterial colonisation and biofilm formation (Giavaresi et al. 2014; Drago et al. 2014; Romanò et al. 2015, 2016; Malizos et al. 2017; De Meo et al. 2021; Zoccali et al. 2021; Carpa et al. 2022). Additionally, crosslinking approaches have been revisited in order to maintain or improve the physical properties through different crosslinking conditions.

Results from early timepoints indicate that the presence of HA hydrogel seemed to hamper osteoblast proliferation. Nevertheless, the 7-day analysis revealed that the cells do proliferate, but in a slower manner. This may be due to the fact that HA is expected to be less cell adhesive and thus the presence of the hydrogel could interfere with the cell-to-cell interactions. This issue could be resolved by incorporating cell-adhesive peptides or polymers such as gelatine in the hydrogel matrix (Tavakoli et al. 2024). While cells have the ability to remodel their surrounding matrix, the mechanical, structural, and biochemical composition of these surroundings also regulates intracellular and transcellular processes (Ahearne 2014). Also, the comparison to a non-crosslinked hydrogel (Healon 5®) that is approved for clinical use showed an identical proliferation pattern.

The apparent reduction in metabolic activity and proliferation observed when osteoblasts embedded in hydrogels were compared to standard cell cultures in the absence of hydrogel should be interpreted with caution. This is expected, as osteoblasts exhibit different metabolic behaviour in a three-dimensional, viscous hydrogel environment compared with standard 2D cultures. The hydrogel itself influences nutrient diffusion and cell migration, which alters baseline metabolic readouts. Importantly, under all conditions where cells were cultured within the hydrogel, both metabolic activity and proliferation were preserved across the tested antibiotic concentrations, supporting the cytocompatibility of the system.

The growth inhibition of *S. aureus* and *S. epidermidis*, as measured by the zone of inhibition following local application of cefuroxime- and vancomycin-loaded hydrogels, further supports the release profile results. The release studies demonstrated a rapid and nearly complete release of cefuroxime, in contrast to a slow release of vancomycin. The significantly smaller inhibition zone observed with 30 × CRC vancomycin, in comparison to 30 × CRC cefuroxime on *S. aureus*, may reflect a lower antibiotic concentration diffusing from the hydrogel, as a substantial amount of vancomycin remained entrapped within the material. Furthermore, the limited growth inhibition of *S. epidermidis* following vancomycin application may be explained by this reduced release. Another contributing factor to this observation could be heterogeneity in vancomycin susceptibility allowing some colonies to grow but not as many. By contrast, cefuroxime was not expected to be effective against *S. epidermidis*, due to the presence of the *mecA* gene associated with *β*-lactam resistance.

### Limitations and strengths

While this study offers valuable insights, certain limitations must be acknowledged. The use of SaoS-2 cells, despite their practicality and ease of culture particularly in the case of screening for suitable concentrations and technical refinements of experimental settings, does not replicate the behaviour and attributes of primary osteoblasts. Furthermore, antibiotic release kinetics were analysed in phosphate-buffered saline (PBS) which does not accurately mimic the complex biochemical and mechanical environment of joint fluid or soft tissues, potentially affecting the degradation and release profiles. Additionally, osteogenic activity is influenced by numerous patient-specific factors, including age, gender, hormonal status, and lifestyle, which were not accounted for in this study. Lastly, the inherent simplicity of in vitro models fails to capture the complexity of in vivo conditions, limiting the direct clinical applicability of the findings (Ghallab 2013).

Various techniques have been described for the isolation of cells with osteogenic properties (Peck et al. 1964; Smith et al. 1973; Jones and Boyde 1977). Additionally, attempts have been made to isolate cells from numerous anatomical sites (Bard et al. 1972; Voegele et al. 2000; Declercq 2004). The literature supports the isolation of human osteoblast cells obtained by enzymatic treatment for in vitro experiments (Regenerative Medicine Institute, National Centre for Biomedical Engineering Science, National University of Ireland, Galway et al. 2012). In our series of experiments, osteoblasts have been obtained from human and mice trabecular bone explants by enzymatic digestion. The addition of SaoS-2 cells provided a cost-effective unlimited supply of material that moreover serves the ‘3R principles’ (replacement, reduction, and refinement). The differences observed between the osteogenic cell types can be explained by differences in their biological characteristics. HoBs displayed robust tolerance to antibiotic exposure, likely reflecting their mature primary cell phenotype and lower baseline proliferation. MoBs showed similar metabolic resilience but differed in proliferation dynamics, which may relate to species-specific signalling pathways and differences in growth kinetics. In contrast, SaOS-2 cells were more sensitive in terms of proliferation, consistent with their osteosarcoma-derived origin, higher metabolic activity, and known vulnerability to environmental stress. These distinctions highlight the relevance of primary human osteoblasts as the most meaningful model when evaluating biomaterial cytocompatibility for orthopaedic applications.

Although biofilm formation is a key pathogenic mechanism in prosthetic joint infections, the present study did not include experiments on the antibiofilm activity of the antibiotic-loaded HA hydrogels. Evaluating effects on mature or developing biofilms requires a distinct experimental design involving prolonged incubation periods, dedicated biofilm quantification assays (such as crystal violet biomass staining, confocal microscopy, or viable cell recovery), and standardised biofilm models specific to *Staphylococci*. As our work focused on material development, cytocompatibility, and initial antimicrobial activity, antibiofilm testing lies beyond the scope of the current study. Future studies will be needed to determine whether this hydrogel system can effectively disrupt or prevent biofilm formation in clinically relevant settings.

Despite these limitations, the study has some strengths. To our knowledge, it is the first to systematically evaluate the toxicity and efficacy of various antibiotic concentrations, with and without HA hydrogels, across multiple osteogenic cell types. The study demonstrates that vancomycin and cefuroxime at concentrations significantly exceeding the CRC for common pathogens are non-toxic to osteoblasts. Notably, cefuroxime at concentrations up to 30 × CRC not only avoids cytotoxicity but also promotes osteoblast proliferation and differentiation, corroborating findings from earlier research (Edin et al. 1996; Salzmann et al. 2007; Rathbone et al. 2011). The inclusion of HA hydrogels further highlights their promise as carriers for local antibiotic delivery. The hydrogels successfully integrated antibiotics and provided controlled release, with cefuroxime exhibiting rapid release and vancomycin requiring higher loading concentrations due to slower release kinetics. That was evident in direct release experiments where the actual presence of the antibiotic was measured by spectrometry, and it was consistent with later bacterial growth inhibition tests. Importantly, the HA hydrogel did not inhibit osteoblast proliferation, which is consistent with previous observations on fibroblast behaviour (Oommen et al. 2013) and comparable to outcomes observed with Healon 5®.

## Conclusions

This is the first study that evaluates the toxicity of various antibiotic concentrations or antibiotic-loaded HA hydrogels on three different osteogenic cell types. The findings demonstrate that both antibiotics, even significantly above clinically relevant concentrations, are non-toxic to osteoblasts. Cefuroxime, in particular, supports osteoblast proliferation and differentiation at high concentrations, while vancomycin requires optimization for sustained release. The HA hydrogel itself proved to be a cytocompatible and safe carrier, offering significant advantages in preventing bacterial proliferation. These findings underscore the potential of utilising antibiotic-loaded hydrogels to achieve localised antimicrobial effects without compromising osteoblast function, offering a promising approach for orthopaedic applications where simultaneous infection prevention and bone healing are critical. Further studies are planned to examine the interactions between the HA hydrogel and various implant surfaces and bacterial colonies, along with the effect of the HA hydrogel on biofilm formation.

## Data Availability

The authors declare that the data supporting the findings of this study are available within the paper. Should any raw data files be needed in another format they are available from the corresponding author upon reasonable request.
